# Spatio-temporal inhabitation of settlements by *Hystrix cristata* L., 1758

**DOI:** 10.1038/s41598-022-09501-5

**Published:** 2022-03-31

**Authors:** Francesca Coppola, Stefano Grignolio, Francesca Brivio, Dimitri Giunchi, Antonio Felicioli

**Affiliations:** 1 Department of Veterinary Sciences, University of Pisa, Viale delle Piagge 2, 56124 Pisa, Italy; 2 Department of Veterinary Medicine, University of Sassari, Via Vienna 2, 07100 Sassari, Italy; 3 Department of Life Science and Biotechnology, University of Ferrara, Via L. Borsari 46, 44121 Ferrara, Italy; 4 Department of Biology, University of Pisa, Via Luca Ghini 13, 56123 Pisa, Italy

**Keywords:** Ecology, Behavioural ecology, Conservation biology

## Abstract

Settlements are usually shared at different times by semi-fossorial mammals. Porcupine reproductive pair shows high den-site fidelity, but no data are available on the spatio-temporal inhabitation of settlements. In this investigation, the spatio-temporal inhabitation of settlements by crested porcupine families was investigated using camera-trapping as well as the ethological factors affecting the settlements selection. The crested porcupine resulted to be the main inhabitant of settlements surveyed in the present study. Each settlement was inhabited exclusively by one porcupine family. Five out of six porcupine families, each alternatively and complementarily inhabited the same two settlements. In all the five monitored families, settlements selection doesn’t follow a seasonal pattern. Settlement inhabitation of porcupines resulted positively affected by cohabitation with badger, while presence of porcupettes did not affect settlements selection. Long periods of settlement inhabitation were positively affected both by the presence of porcupettes and cohabitation with badger. The pattern of settlements inhabitation in relation to their availability and porcupine population density as well as factors promoting porcupine-badger cohabitation should be further investigated. New ethological knowledge obtained in this investigation could be involved in the evaluation of the ecological epidemiology of infectious diseases between porcupine and badger within a one health approach and may be a useful tool for a sustainable management of semi-fossorial mammals.

## Introduction

Seasonal variation in the pattern of animal use of space may be linked to factors such as food availability, vegetative cover, habitat structure, predation, and environmental temperature that play a key role in survival and reproduction^1–7^. Semi‐fossorial rodents dig underground burrows for breeding and protection from predators and environmental stress^8^ while they spend time outside the burrow to forage, defend territories, and seek out potential mates^9^. The spatio-temporal inhabitation of burrow systems by semi-fossorial mammals has been a neglected topic even though it is a key aspect for the: (I) assessment of rodent ecology and population dynamics and (II) conservation and management of rodents in a human-modified ecosystem where these species may cause conflicts with human activities (i.e., burrow digging in riverbanks or dams and damage to drainage pipes). To the best of our knowledge, there is a lack of data concerning the seasonal pattern of burrow inhabitation as well as the inhabitation of different settlements. In *Microtus pinetorum*, *Rhombomys opimus*, *M. arvalis* and *Octodon degus* relations between animal density, burrows abundance and their utilization were investigated^7,10–13^. In *Octodon degus*, burrow systems’ availability seems not to be linked to population density and no association was recorded between animal density and number of burrows occupied^13^. Conversely, a marked seasonal and spatial pattern in both burrow density and abundance of occupied burrows was recorded in *M. arvalis*^7^.

The crested porcupine (*Hystrix cristata*) is a large semi-fossorial, mainly nocturnal rodent^14^. It is gregarious and lives in families composed by the adult reproductive pair, porcupettes (< 5 months old), juveniles (from 5 to 8 months old) and sub-adults (from 8 to 12 months old)^14–16^. The porcupine locomotor activity is light-dependent and shows a rhythmic variation well synchronized to the synodic lunar cycle^17,18^. The nocturnal motor activity is more intense during the new-moon phases and decreases during the full-moon phases (moonlight avoidance)^17,19^. The crested porcupine also shows daytime locomotor activity^19,20^, often performing sunbathing^21^. The crested porcupine spends most of the nocturnal hours searching for food and for social activities within the family^17,22^, and the burrow is the main site for breeding, sleeping and refuge during the daylight hours^18,20^. Seasons did not affect crested porcupine reproduction and porcupettes birth occurs throughout the year^23^. Porcupettes are born in burrow and their first emergence from burrow occur from 10 to 15 days after birth^23^. Previous studies indicate that only one reproductive pair of porcupines inhabit a settlement showing a great site fidelity^14,24^. The use of more than one settlement by a porcupine pair was hypothesised by observing the porcupine signs of presence (i.e., quills and footprint)^17,18^. A seasonal preference for different burrow orientations throughout the year has also been hypothesised^18^. In winter porcupines seems to choose south-oriented burrows, in summer those north-west oriented while in spring and autumn no preference was reported^18^. Based on the above hypothesis the use of different settlement throughout the year was suggested^18^.Settlements (i.e., cluster of ground entrance holes) inhabited by crested porcupine are usually also inhabited or explored, at different times (i.e., settlement sharing), by badger (*Meles meles*) and red fox (*Vulpes vulpes*)^22,25^, even if cohabitation (i.e., simultaneously inhabitation of the same settlement) has been observed only between porcupines and badgers^25^. Cohabitation between porcupine and badger occurs throughout the year even in presence of porcupettes^25^. The simultaneous inhabitation of the same settlements by porcupine and badger is not due to lack of settlements availability and often resulted in an increase of aggressive interaction between the two species^25^. However, no data are available concerning how porcupine-badger cohabitation and porcupettes presence can affect porcupine settlement selection and inhabitation as well as whether cohabitation causes the abandonment of a settlement by a porcupine family.

No other information is available in the scientific literature concerning the inhabitation of available settlements in the territory by crested porcupine families. There is also a lack of evidence supporting the hypothesis that the same porcupine family may inhabit different settlements throughout the year and if this behavioural pattern may be affected by the presence of porcupettes or co-habitation with other semi- fossorial mammals. Therefore, this study aims to investigate the spatio-temporal inhabitation of the available settlements by crested porcupines using camera-trapping and how ethological factors as porcupettes presence and co-habitation with badgers can affect their selection.

## Results

Overall, 10 porcupines were captured, marked, and made individually recognisable. Moreover, six no-marked individuals were individually identified due to the presence of phenotypic peculiarities (see Supplementary Table S1). Based on individual recognition of marked and phenotypic peculiar individuals, six porcupine families were identified. The average size of the family was 4.1 ± 1.5 (mean ± SD) individuals.

### Spatio-temporal inhabitation of settlements

**Figure 1:**
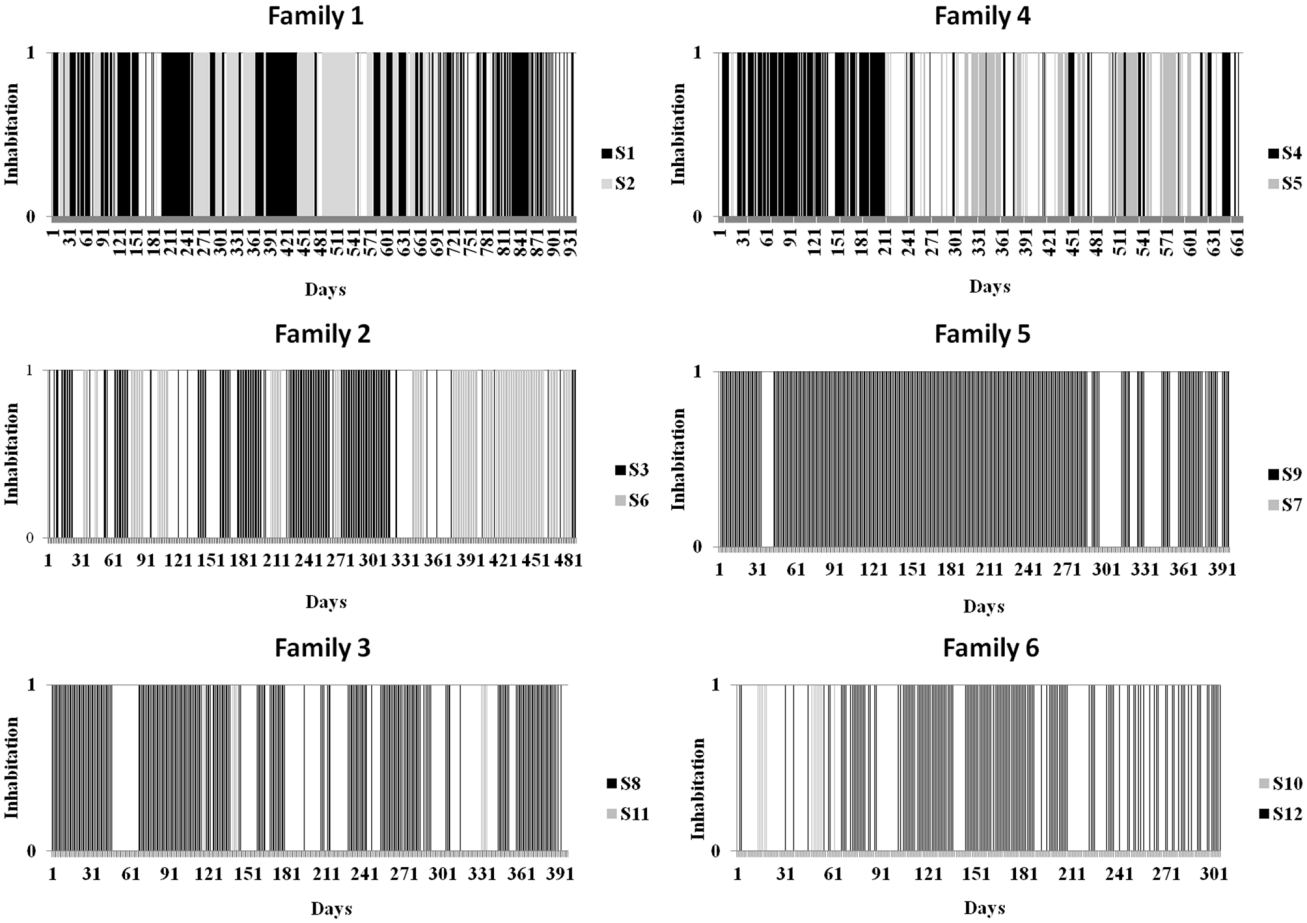
Inhabitation pattern of each porcupine family in the two inhabited settlements (black and grey coloured) during the whole monitoring period. Black colour always refers to the main inhabited settlement. White spaces indicate the days in which it was not possible to assess inhabitation.

Among the 68 monitored settlements 69% (n = 47) resulted inhabited by porcupines while 31% (n = 21) resulted abandoned and/or only occasionally visited by porcupines, badgers, or red foxes at different times. The minimum time of permanence of porcupine in burrow during daylight hours resulted 10 h and 11 min, the maximum 16 h and 41 min with an average time of 13 h and 44 min (SD = 1 h and 26 min). Each experimental settlement was inhabited by a single and recognisable porcupine family and no cohabitation among other porcupine families was detected. In four occasions, two in settlement S1 and one in settlements S2 and S5, encounters between resident porcupines (i.e., porcupines inhabiting the settlement) and porcupines visiting the settlement were recorded. In all these encounters, fighting between individuals belonging to different families were recorded and the resident family continued to live in the settlement after the fighting. During the whole period of monitoring, five out of six porcupine families each have alternatively and complementarily inhabited the same two settlements, which were no more than 250 m from each other. Only one family (family 5) inhabited a single settlement. The inhabitation of two settlements by another family of porcupines other than the “owner” was never observed even when the “resident” family was not present. Each porcupine family showed a different inhabitation pattern of the two inhabited settlements during the whole period of monitoring (Table 1, Fig. 1). Only family 5 always inhabited a single settlement and no inhabitation was recorded in the other one monitored. A different inhabitation pattern of the two settlements was also recorded during the same period in the 5 porcupine families simultaneously monitored (see Supplementary Fig. S1). In family 1 and family 4 different inhabitation patterns of the two settlements in subsequent years of monitoring was recorded (see Supplementary Fig. S2).

**Table 1:** Inhabitation frequencies (days of inhabitation (n)/total days of monitoring) of each porcupine family in each settlement during the whole investigation period.

	Settlements	Inhabitation frequency
Family 1	S1	58.6% (n = 421)
	S2	41.4% (n = 297)
Family 2	S3	54.7% (n = 181)
	S6	45.3% (n = 150)
Family 3	S8	95.2% (n = 239)
	S11	4.8% (n = 12)
Family 4	S4	58.2% (n = 230)
	S5	41.8% (n = 165)
Family 5	S9	100% (n = 331)
Family 6	S10	7% (n = 12)
	S12	93% (n = 158)

**Figure 2:**
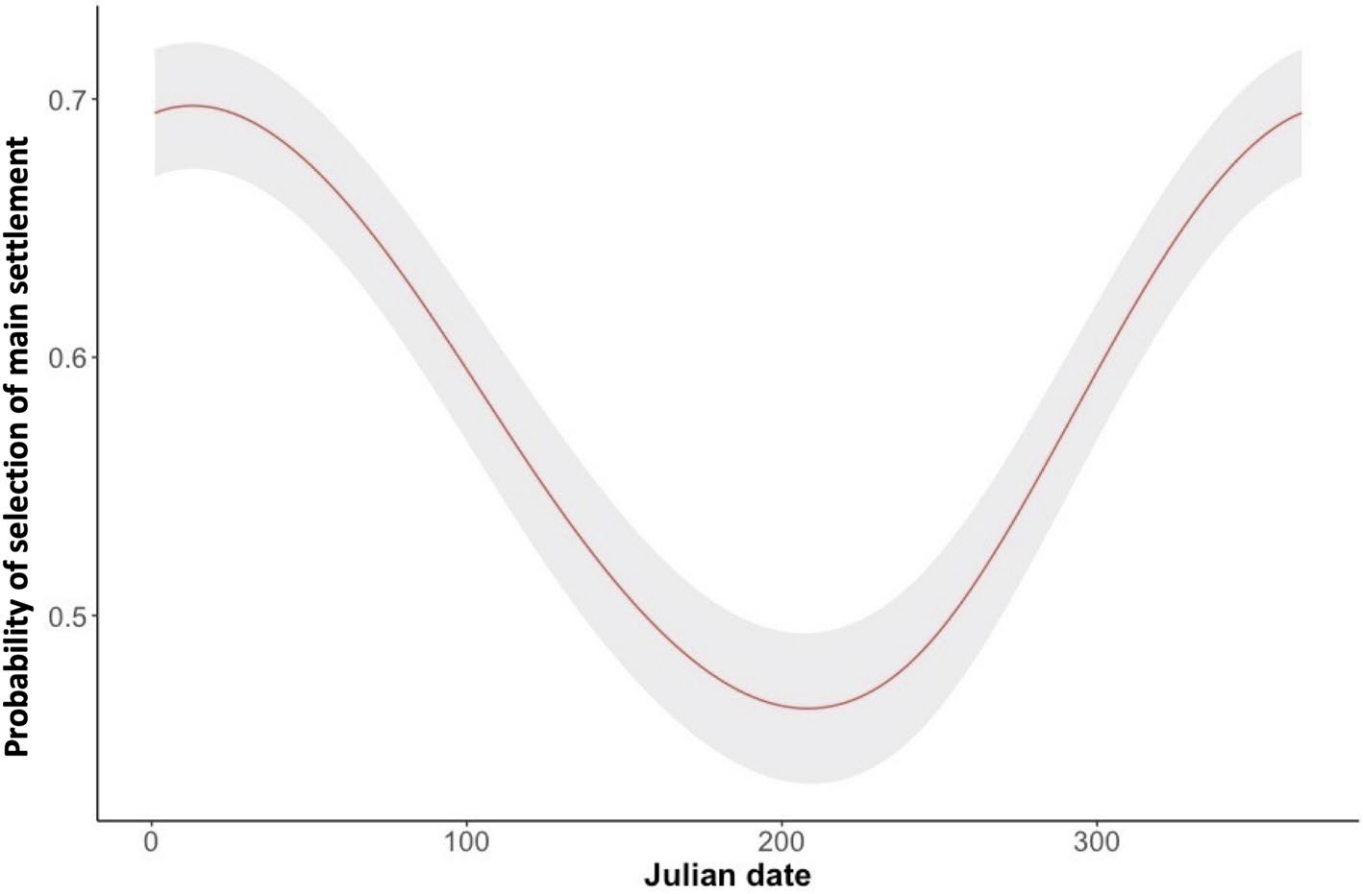
Probability of selection of the settlements (main vs secondary) by crested porcupine in Central Italy as predicted by the Generalised Additive Model (with a binomial error distribution) including reproductive status, cohabitation, Julian day, and family ID as predictive variables, sample size = 2221. The figure shows the effects exerted by Julian date. The predictions are given setting reproductive status = 1, cohabitation = 0, and family ID = 1. In the graph the colour-shaded areas are the estimated standard errors.

Throughout the year, the probability to select the main settlement peaked around 13 January (i.e., 13th Julian day), while minimum values were recorded around 27 July (i.e., 208th Julian day, Fig. 2). Cohabitation with badger increased the probability to inhabit the main settlement, whereas this was not affected by the reproductive status (i.e., the presence of porcupettes—Table 2).

**Table 2:** Effect of predictor variables (reproductive status, cohabitation, Julian day, and family ID) estimated by the Generalised Additive Model (with a binomial error distribution) fitted to predict the inhabitation of the settlements (main vs secondary) by crested porcupine in Central Italy.

	Estimate	Std. error	z value	p-value
**Parametric coefficients**				
(Intercept)	1.120	0.719	1.557	0.1194
Reproductive status	0.012	0.108	0.107	0.915
Cohabitation	2.478	1.030	2.406	0.016

	edf	Ref.df	F	p-value
**Approximate significance of smooth terms**				
s (Julian day)	1.923	2.000	187.7	< 0.001
s (family)	3.944	4.000	108.2	< 0.001

Coefficients (Estimate) for the linear terms in the model (reproductive status, cohabitation), standard errors (Std. Error), test statistic and its significance (z value and p-value) are shown in the first part of the Table. Effective degrees of freedom (edf, representing the complexity of the smooth terms), together with test statistic (F), its significance (p-value) and degree of freedom (Ref.df) used to assess overall significance of the smooth terms, are shown in the second section.

**Figure 3:**
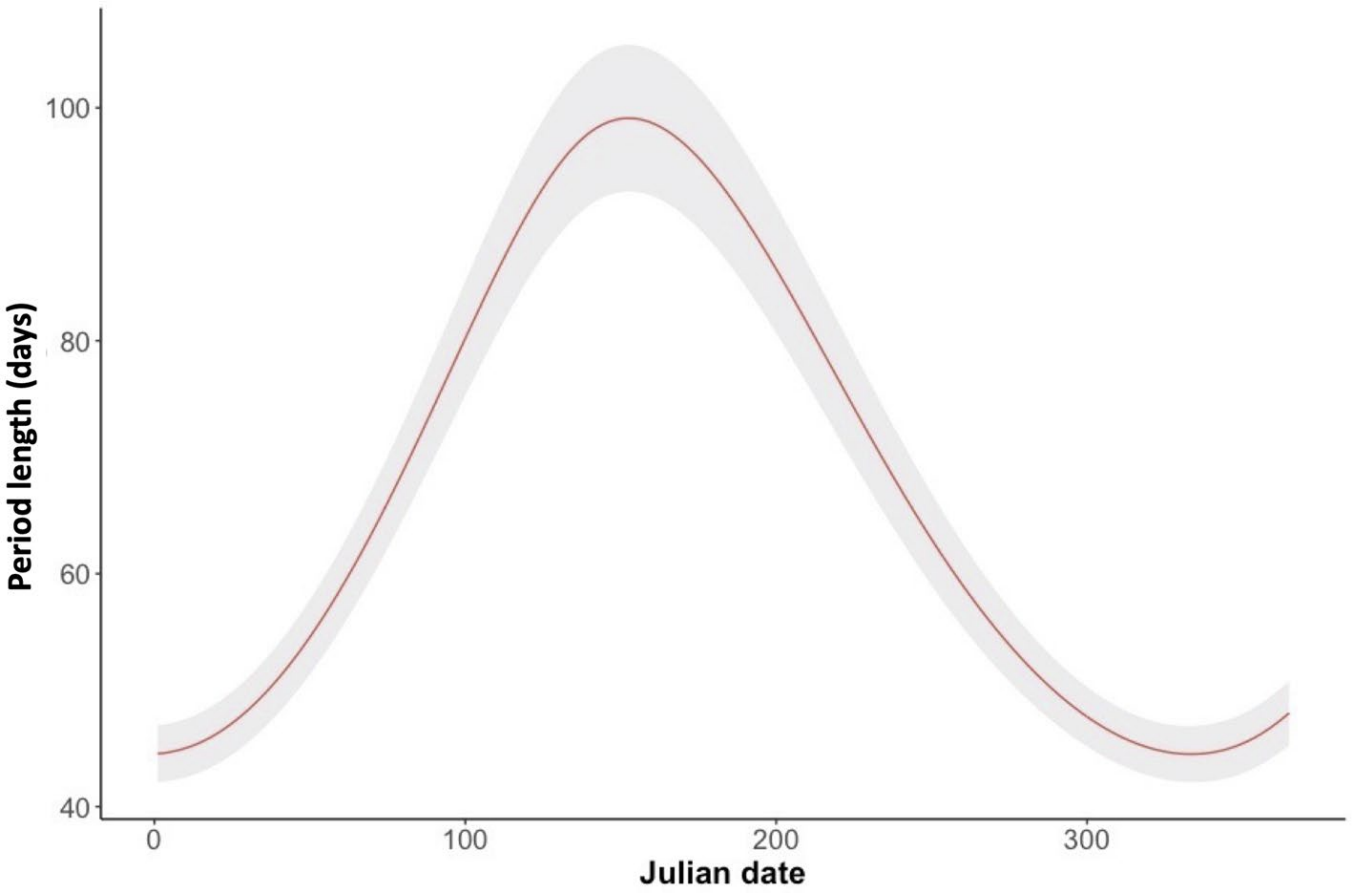
Values of inhabitation period length (days) of a settlement by porcupine in the province of Pisa (Italy), as predicted by the Generalised Additive Model (with a Poisson error distribution) including settlement, reproductive status, Julian day, and family ID as predictor variables, sample size = 2221. The figure shows the effects exerted by Julian date. The predictions are given setting settlement = main, reproductive status = 1, cohabitation = 0, and family ID = 1. In the graph the colour-shaded areas are the estimated standard errors.

The inhabitation of the settlements by each family ranged from 1 to 244 consecutive days with an average time of permanence in each settlement of 8.1 ± 17.8 days. In 24 occasions long periods of permanence in the same settlement (> 20 consecutive days) were recorded and in 50% of these (n = 12) porcupettes were present. Overall, 133 events of settlement change were recorded. In 2.3% (n = 3) of settlements change events badger or red fox exploratory activity in the previous days were recorded while in the remaining cases (n = 130, 97.7%) the change occurred without any detectable source of disturbance. The period length of inhabitation by a family in a settlement varied throughout the year (Table 3). At the beginning of summer (pick on 2 June—153th Julian day) PL was more than double (99.1 versus 44.5 days) that at the beginning of winter (29 November—333th Julian day; Fig. 3). The porcupine families inhabited for longer periods a settlement (main settlement) than the secondary one (Table 3). The presence of porcupettes, and cohabitation with badgers positively affected PL (Table 3).

**Table 3:** Effect of predictor variables (settlement, reproductive status, cohabitation, Julian day, and family ID) estimated by the Generalised Additive Model (with Poisson error distribution) fitted to predict the length of inhabitation period by porcupine families in a settlement in Central Italy.

	Estimate	Std. error	z value	Pr( >|t|)
**Parametric coefficients**				
(Intercept)	1.724	0.100	17.202	< 0.001
Settlement (main/secondary)	0.252	0.049	5.122	< 0.001
Reproductive status	0.896	0.056	15.879	< 0.001
Cohabitation	1.360	0.059	23.184	< 0.001

	Edf	Ref.df	F	p-value
**Approximate significance of smooth terms**				
s (Julian day)	1.944	2.000	621.4	< 0.001
s (family)	3.585	4.000	60.9	< 0.001

Coefficients (Estimate) for the linear terms in the model (settlement, reproductive status, cohabitation), standard errors (Std. Error), test statistic and its significance (z value and p-value) are shown in the first part of the Table. Effective degrees of freedom (edf, representing the complexity of the smooth terms), together with test statistic (F), its significance (p-value) and degree of freedom (Ref.df) used to assess overall significance of the smooth terms, are shown in the second section.

## Discussion

The behavioural pattern of settlements inhabitation by the semi-fossorial rodent *H. cristata* was here investigated for the first time. The crested porcupine inhabited the 69% of monitored settlements, occasionally cohabiting with badger while red fox was only occasional visitor of these settlements. This allows us to ascribe the crested porcupine as main inhabitant of investigated settlements. The findings obtained in this investigation show that each settlement is inhabited by a single porcupine family and cohabitation between more porcupine families has never been documented. This result confirms the high burrow fidelity by crested porcupine previously recorded^24^. In this investigation encounters between porcupines that inhabited the settlement and porcupines visiting the same settlement were recorded in four occasions and aggressive interactions or fighting always followed. On all occasions, the occurring of fighting did not lead the resident porcupine family to leave the inhabited settlement. These observations allow us to hypothesise that crested porcupine families use exclusive territory, and the occurrence of fighting is probably due to settlement defence. Territorial behaviour in porcupine was only observed in semi-captive crested porcupine^17^ and in captive cape porcupine (*Hystrix africaeaustralis*)^26,27^. In wild cape porcupines the presence of territoriality was also hypothesised^27^. The territoriality in free-ranging crested porcupines has never been demonstrated^28,29^ and some Authors refer to *H. cristata* as a non-territorial rodent^30^. However, the observations performed in this study suggest the presence of an intra-specific territory defence in the crested porcupine and further investigation is necessary.

Results obtained show that most of the monitored porcupine families did not inhabit a single settlement, but rather inhabited two different settlements. The 83.3% (5 out of 6) of the monitored porcupine families alternatively and complementarily inhabited the same two settlements during the whole period of monitoring confirming what previously hypothesised by Felicioli^17^ and Felicioli and Santini^18^. The exclusive inhabitation of both settlements by a single porcupine family suggests that porcupine settlements inhabitation may be distributed in a spatio-temporal binary system. The two settlements inhabited by each monitored porcupine family has never been inhabited by other porcupine families not even when the resident family was not present. This result potentially suggests that territorial defence may not be strictly linked to the main inhabited settlement, but it extends to the secondary one. Moreover, the exclusive inhabitation of a binary system by each porcupine family indicates that settlements availability does not necessarily reflect porcupine population density. At the same time binary settlements availability may be affected by porcupine population density. No information is available concerning porcupine population density in our study area; however, it could be interesting to assess whether the same pattern of settlements inhabitation recorded in this study is also detectable in areas of higher porcupine density than in our study area. To the best of our knowledge no data are available on the spatio-temporal burrow inhabitation by semi-fossorial rodents and very little is known concerning the relationship between rodent population trends and burrow availability. Abundance and quality of burrows were hypothesised to be limiting factors in the population dynamics of rodents such as *Microtus pinetorum* and *Rhombomys opimus* for which underground burrows are essential for breeding^11,12^. Conversely, in *Octodon degus* population density seems not linked to burrow systems availability and no association was recorded between animal density and number of used burrows^13^. Mackin-Rogalska et al.^10^ suggested an important role of burrow systems in generating multi-annual cycling in the *M. arvalis* population trend. In this species, a marked seasonal and spatial pattern in both burrow density and abundance of occupied burrows was recorded^7^. Investigation concerning settlements inhabitation in relation to their availability and porcupine density are desirable.

The results obtained in our study also show that each family exhibited a different inhabitation pattern of the two settlements, during the whole period of monitoring, selecting a main settlement. The probability to inhabit the main settlement was affected by Julian date and, surprisingly, increased when badgers were present, whereas it was not affected by the presence of porcupettes. Results obtained in this investigation show that settlements inhabitation by crested porcupine does not follow a seasonal pattern. The different inhabitation pattern of one settlement in consecutive years suggests that settlements selection may be due to different characteristics in architecture or environmental conditions (e.g., exposition). In the present study, due the small sample size, does not allow to confirm the occurrence of porcupine seasonal preference due to settlement exposition as previously reported in other studies^17,18^. Further studies should aim to investigate any potential characteristic which may affect the seasonal inhabitation of different settlements by this species.

Among investigated ethological factors, potentially affecting settlements selection, cohabitation with badger positively influenced the settlement inhabitation by porcupines. This result suggests the absence of porcupine-badger competition. Occurrence of co-habitation between porcupine and badger is not due to a lack of settlement availability or to the absence of aggressive interactions that frequently occurred between the two species in the same study area^25^. Moreover, cohabitation with badger positively affected the length of the inhabitation periods in a settlement by a porcupine family. These findings are surprising and suggests that there is not an inter-specific competition between the two species and that they can have potential mutual advantages in cohabitating. Therefore, further investigations to assess the factors (e.g., settlements features, number of chambers and animal density) promoting the occurrence of cohabitation as well as the potential advantages of cohabitation for both species are needed. Results obtained in this investigation also show that the presence of porcupettes does not affect the selection of settlements for inhabitation by crested porcupine. In Indian crested porcupine (*Hystrix indica*) the internal architecture of the burrow as well as the settlements use by other burrow dwelling vertebrates has been reported to affect settlement occupancy^31,32^. Although in crested porcupine the birth of porcupettes occurs indifferently in both settlements, the above factors may still have a role in the selection of the settlements by the breeding female and further investigation is needed. Burrow use for breeding is an important component in the ecology of several mammal species to limit the risk of predation on juveniles and increase their survival probability^33–36^. Environmental features (i.e., food availability, temperature, humidity) as well as social factors, such as the presence of competitors and predators, are likely to affect burrow use in some rodent species^37,38^. In particular, in eastern chestnut mouse (*Pseudomys gracilicaudatus*) seasonality in refuge site use seems to reflect the reproductive and environmental trade-offs when resources are scarce^38^. In crested porcupine seasonality does not affect reproduction, which occurs throughout the year^23,39^. Noteworthy, in Italy crested porcupines do not have natural predators able to kill healthy adult specimens^24,40^, even if the role of porcupine in wolf diet needs to be clarified. Predation on porcupettes is made difficult by effective and constant protection by at least one adult^15,39^. The length of inhabitation period by a family in a settlement resulted positively affected by the presence of porcupettes. First emergence of porcupettes from burrow occurs at 10–15 days after birth and during the first 20 days after the first emergence from burrow they always remain in the surrounding of the settlement^23,39^. Porcupettes start to follow the adult and increasingly move further away from the burrow when they are about one month old^23^. Therefore, the result obtained in this investigation is not surprising and it is most probably linked to porcupettes rearing. In conclusion the results achieved in this investigation provide new ethological knowledge on porcupine spatio-temporal inhabitation of settlements that can be a base for further investigation on ecological factors driving settlements selection. Investigation on trend in settlements inhabitation in relation to their availability, environmental conditions, and density of porcupine and badger population are desirable. In addition, further investigations to assess the factors promoting cohabitation as well as the potential advantages of cohabitation for both species are needed. Results obtained in this investigation could be involved in the evaluation of the ecological epidemiology of infectious diseases between porcupine and badger within a one health approach. Moreover, these results may be a useful tool for the sustainable management of the impact of these semi-fossorial mammals on human infrastructures such as riverbanks.

## Materials and methods

### Study area

**Figure 4:**
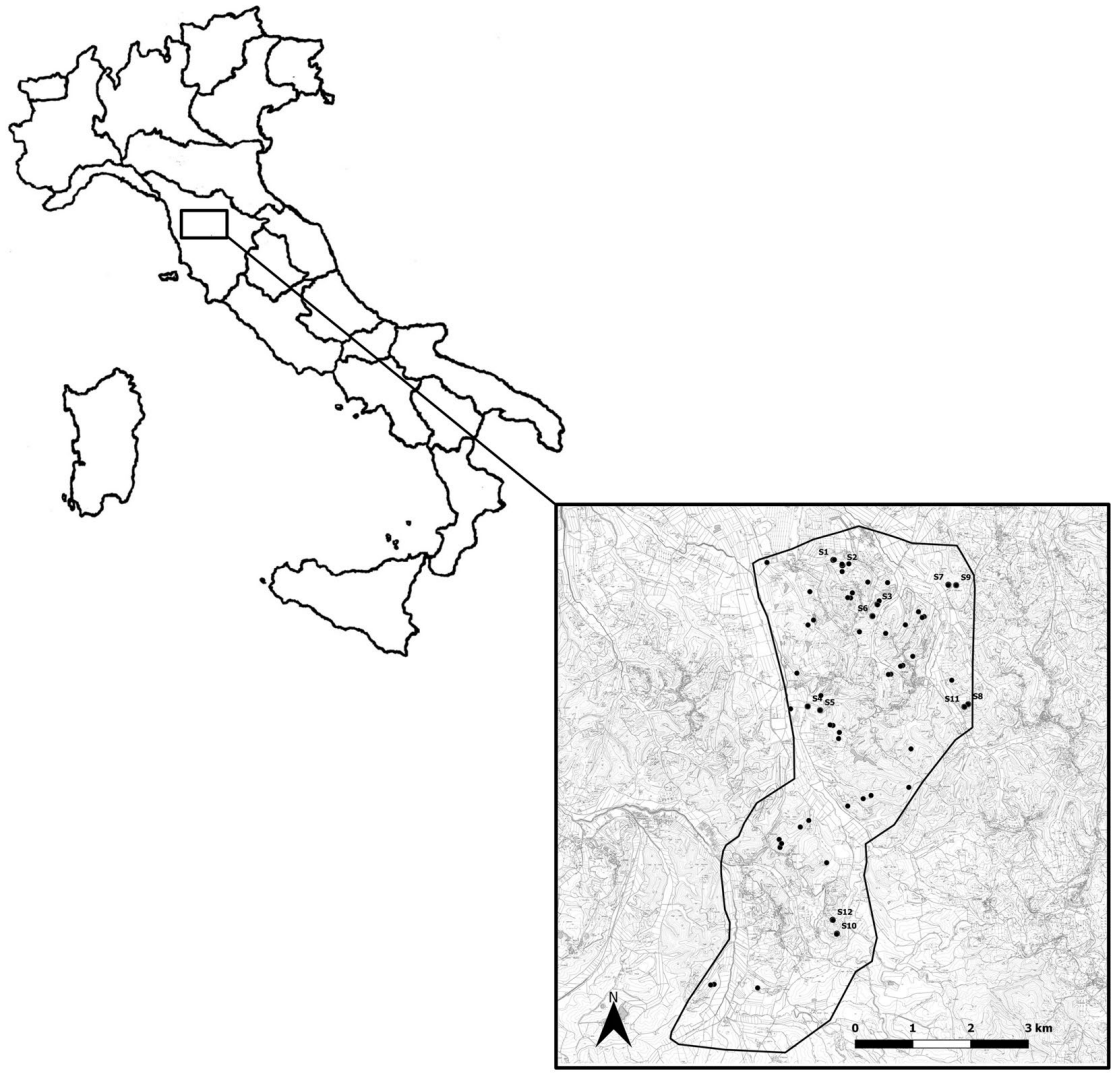
Map of Italy: in the inset detail of the study area (border black line), known and monitored settlements for porcupine’s inhabitation frequencies analysis (black dots) and the monitored experimental settlements (black dots, from S1 to S12) where the investigation on the spatio-temporal inhabitation were performed.

The collection of data was performed in a hilly area (86 m a.s.l.) of 2583 ha in the municipality of Crespina-Lorenzana (43.35412° N; 10.325052° E) in the province of Pisa (Tuscany, Central Italy) (Fig. 4). The study area is characterized by a high biodiversity and environmental fragmentation in which small woody areas are interspersed with both non-cultivated and cultivated areas and rivers. The woody cover is characterized by deciduous forest, mainly of *Robinia pseudoacacia*, *Quercus cerris*, *Q. pubescens* and *Q. ilex* with a thick and thrive undergrowth. The undergrowth is composed by a large variety of shrubs and herbaceous plants such as *Sambucus nigra*, *Rubus ulmifolius*, *Laurus nobilis*, *Ruscus aculeatus*, *Juniperus oxycedrus*, *Asparagus acutifolius*, *Cyclamen* sp., *Tamus communis* and *Orchis provincialis*. The climate is temperate with hot summers (average 23.1 °C in August) and rainy and mild winters (average 6.8 °C in January). The average annual rainfall is 842 mm with peaks between October and December (https://it.climate-data.org/europa/italia/tuscany/crespina-110310/). In the study area 68 settlements were known, for a density of 2 settlements/km^2^^41^.

### Capture-marking activity

**Figure 5:**
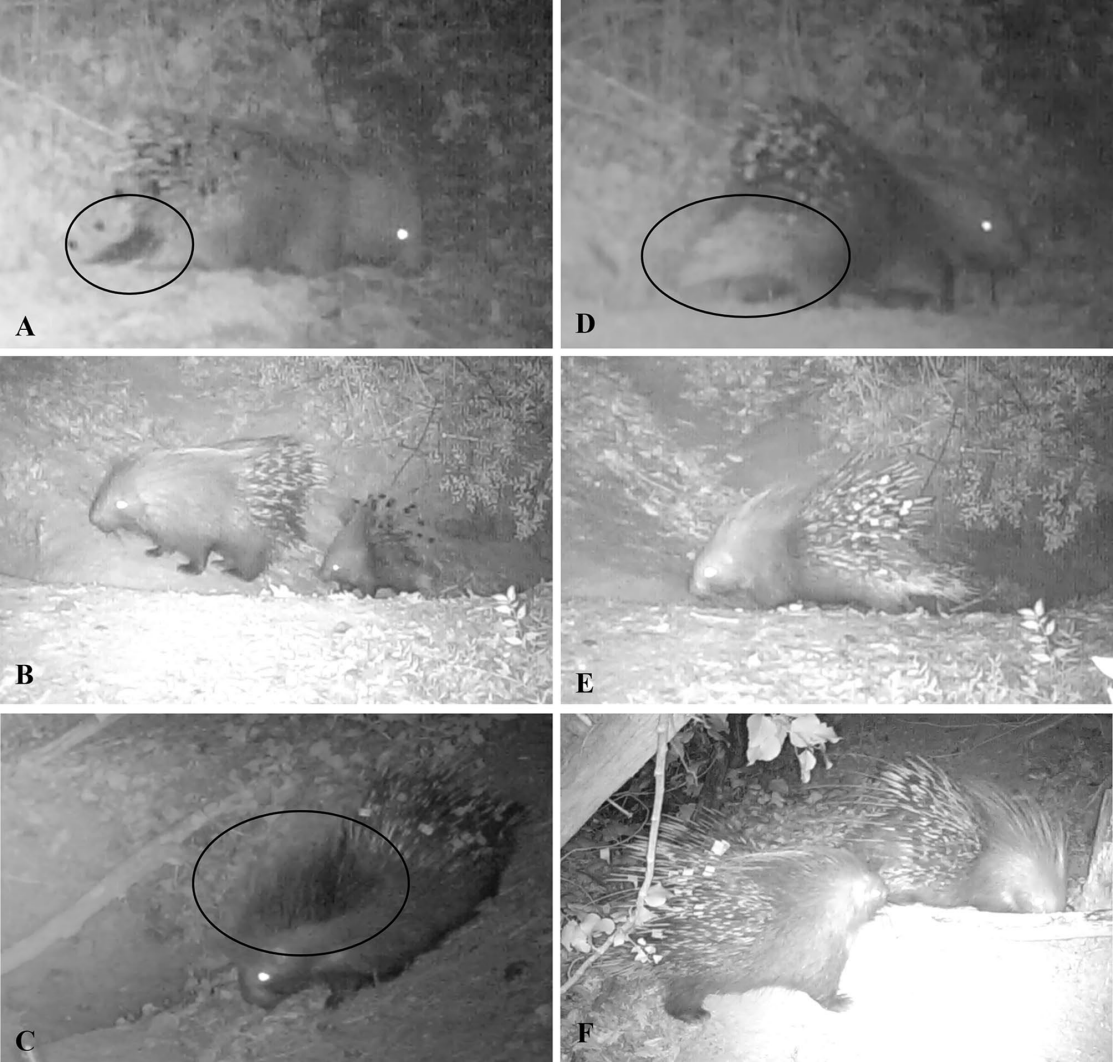
Screenshot of camera traps videos of some marked porcupines. (**A**) Adult male of family 4 in S4 marked with black tapes on quills and black paint on the tail (in the circle). (**B**) Porcupettes female of family 6 in S10 marked with black tape on quills. (**C**) Sub-adult female of family 1 in S1 marked with red tapes (visible as grey tape in greyscale camera trap videos) on quills and black paint on the crest (in the circle). (**D**) Sub-adult female of family 4 in S4 marked with red tapes (visible as grey tape in greyscale camera trap videos) on quills and white paint on the tail (in the circle). (**E**) Sub-adult female of family 6 in S10 marked with white tapes on quills. (**F**) Adult male of family 5 in S9 marked with white tapes on quills.

Between February and March in 2018 and 2019 two porcupine capture sessions were performed to make each captured animal recognisable in the videos recorded by camera-traps. Porcupine capture and handling were carried out in accordance with guidelines and protocol approved by the Italian Institute for Environmental Protection and Research (ISPRA) with protocol number 22584 of 8th May 2017 and by the Tuscany Region with the Decree n. 14235 of 3rd October 2017. Porcupines were captured in wire mesh traps (110 × 42 × 42 cm) baited with corn and potatoes. In each capture session, six traps were placed in the most used porcupine pathways near the settlements and were active for 30 continuous days. Each capture trap was equipped with a numbered identification plate supplied by the Province of Pisa and monitored by a camera-trap. The capture traps were checked two times a day and the no-target species captured were immediately released. Each captured porcupine was weighted, anesthetized, sexed and the age class was estimated based on body weight (Adults > 11 kg, Sub-adults 8–11 kg, Juveniles 5–8 kg, Porcupettes < 5 kg), according to Coppola et al.^21^. Furthermore, the animals were individually marked by applying coloured adhesive tapes on the quills or spraying white or black paint on the crest and/or the tail or by combining these two types of marks (Fig. 5). In addition to the marked porcupines, other individuals were individually recognisable by the presence of phenotypic peculiarities (e.g., mainly blindness and presence of injuries). The presence of marked or individually recognisable animals allowed us to identify the porcupine families. For each recognised porcupine family, the number of individuals was recorded.

### Data collection on the spatio-temporal inhabitation of the settlements

The camera-trapping monitoring was performed between 2016 and 2019. Camera-traps (Num’axes PIE1009) with passive infrared sensor (PIR) were used to assess inhabitation of settlements by crested porcupine. The camera-traps were active for 24 h a day and set to record 20 s video clips without time lapse and to record date and hour in each video recorded.

Experimental settlements were chosen among the 68 known settlements, for a total of 314 ground holes, present in the study area. Each known settlement was previously monitored for a period ranging from 60 to 90 days to assess the effective inhabitation by a porcupine family. Twelve inhabited settlements, identified by using a code from S1 to S12, were used to investigate the trends in spatio-temporal inhabitation by crested porcupine (Fig. 4, see Supplementary Table S2). Each experimental settlement was constantly monitored for different time periods ranging from 10 to 31 months by using two camera-traps per settlement to maximize the number of monitored holes used by porcupines (see Supplementary Table S2). All experimental settlements were in steep sandy soil in deciduous woodlands, within a range of 80 m from either cultivated or non-cultivated areas. The average distance between the settlements was 2.6 km (standard deviation (SD) = 1.4 km) and the minimum distance was 120 m. The distance between the settlements and the environmental characteristics (i.e., presence of gorge, streams, street, path etc.) excluded the presence of any direct underground connection between each pair of settlements. The inhabitation of settlements by crested porcupine was determined by assessing the time of permanence in the burrow in each day of monitoring. The settlements were considered inhabited if the permanence of a porcupine family in the burrow during daytime was confirmed. The permanence was defined as daytime continuously spent by porcupine either inside or in front of the burrow (e.g., performing sunbathing and/or lactation) being longer than the minimum recorded time interval between the last coming in and the first coming out from the burrow^25^. The days when permanence in the settlements could not be determined were excluded from the analysis. For each porcupine family, the number of settlements inhabited, the days of inhabitation in each settlement and the number of settlement changes were recorded. In addition, for every settlement inhabited by a family, the daily presence of porcupettes and occurrence of cohabitation with badger was recorded. The settlements were considered co-habited when at least two individuals of different species simultaneously inhabited the same settlement.

### Statistical analysis

The total number of inhabitation days by a family in each settlement was measured. As each family used a maximum of two settlements (see “Results” section for more details), we translated this information into a binary variable (inhabited settlement). We categorised as “main settlement” the settlement most inhabited by the family and as “secondary settlement” the other one. A dataset consisting of a record per day per family and including information on “inhabited settlement”, Julian date, family ID, and two binary variables reporting the presence of porcupettes (reproductive status) and badgers (cohabitation) on the same day was built. To analyse the inhabitation of the settlements by a family, i.e., the selection between the two settlements used by a family, the binary variable “inhabited settlement” was modelled by using Generalized Additive Model (GAM) with binomial distribution. GAM was implemented within the *mgcv* package in R^42^. In order to account for annual variations in porcupine settlement inhabitation patterns the date (Julian day), was included in the model. The effect of the date was modelled as a cyclic cubic regression spline, to take into account the circularity of this variable: thus, we ensured that the value of the smoother at the far-left point (1 January) was the same as the one at the far-right point (31 December). In the model, reproductive status and cohabitation were also included as explanatory variables. Because of the nested nature of data, we used family identity as a random factor by declaring it in the GAM formula using “re” terms and smoother linkage^43^.

In order to estimate the effects of the explanatory variables on the length of inhabitation period in a settlement by a family, a new variable (period length, PL—number of consecutive days of inhabitation by a family in a settlement) which described the number of consecutive days of inhabitation by a family in a settlement was defined. In each record of the new dataset, the family identity, the PL, the inhabited settlement (main vs secondary), the Julian day when the family started to inhabit the settlement, the reproductive status (porcupette presence *vs* absence) and cohabitation (badger presence vs absence) were included. Porcupettes and badgers were considered as present if we recorded their presence at least one day during the period. Then, we modelled PL by using a GAM with Poisson distribution. Similarly, to the previous model, family identity was included as random factor and the effect of Julian date was modelled as a cyclic cubic regression spline, to take into account the circularity of this variable. The other variables included in the model were the inhabited settlement, the reproductive status and cohabitation.

For each dependent variable (inhabited settlement and PL), to avoid collinearity we checked for possible correlations between continuous predictor variables by calculating Pearson correlation coefficient within all possible predictor variables pairs^44^. Moreover, we screened predictors for multicollinearity by calculating the variance inflation factors (VIF). We found only negligible correlations (r <|0.7|) and no severe multicollinearity between the variables (VIF < 3)^44^, thus all predictors were considered in the analysis. The goodness of fit of the GAMs (e) was checked by visual inspection of residuals^44^.

### Ethical approval

The capture-marking activity of resident porcupines was approved by the ethics committee of Italian Institute for Environmental Protection and Research (ISPRA) with protocol number 22584 of the 8 May 2017 and by Tuscany Region with the Decree n. 14235 of the 3 October 2017 and porcupine capture and handling were performed in accordance with guidelines and protocol approved by these institutions. This study was carried out in compliance with the ARRIVE guidelines.

## Supplementary Information

Supplementary Figure S1.

Supplementary Figure S2.

Supplementary Table S1.

Supplementary Table S2.

## Data Availability

All data are available on request to the corresponding author.
