# Supplementary material for: Spatio-temporal inhabitation of settlements by Hystrix cristata L., 1758
Source: Sci Rep. 2022 Mar 31;12:5426. doi: 10.1038/s41598-022-09501-5 (PMC8971386; doi:10.1038/s41598-022-09501-5)

**Supplementary Figure S1.** Inhabitation patterns of 5 porcupine families in their two inhabited settlements (black and grey coloured) during the same period of monitoring (July 2018 and July 2019). Black colour always refers to the mostly inhabited settlement. White spaces indicate the days in which it was not possible to assess inhabitation.


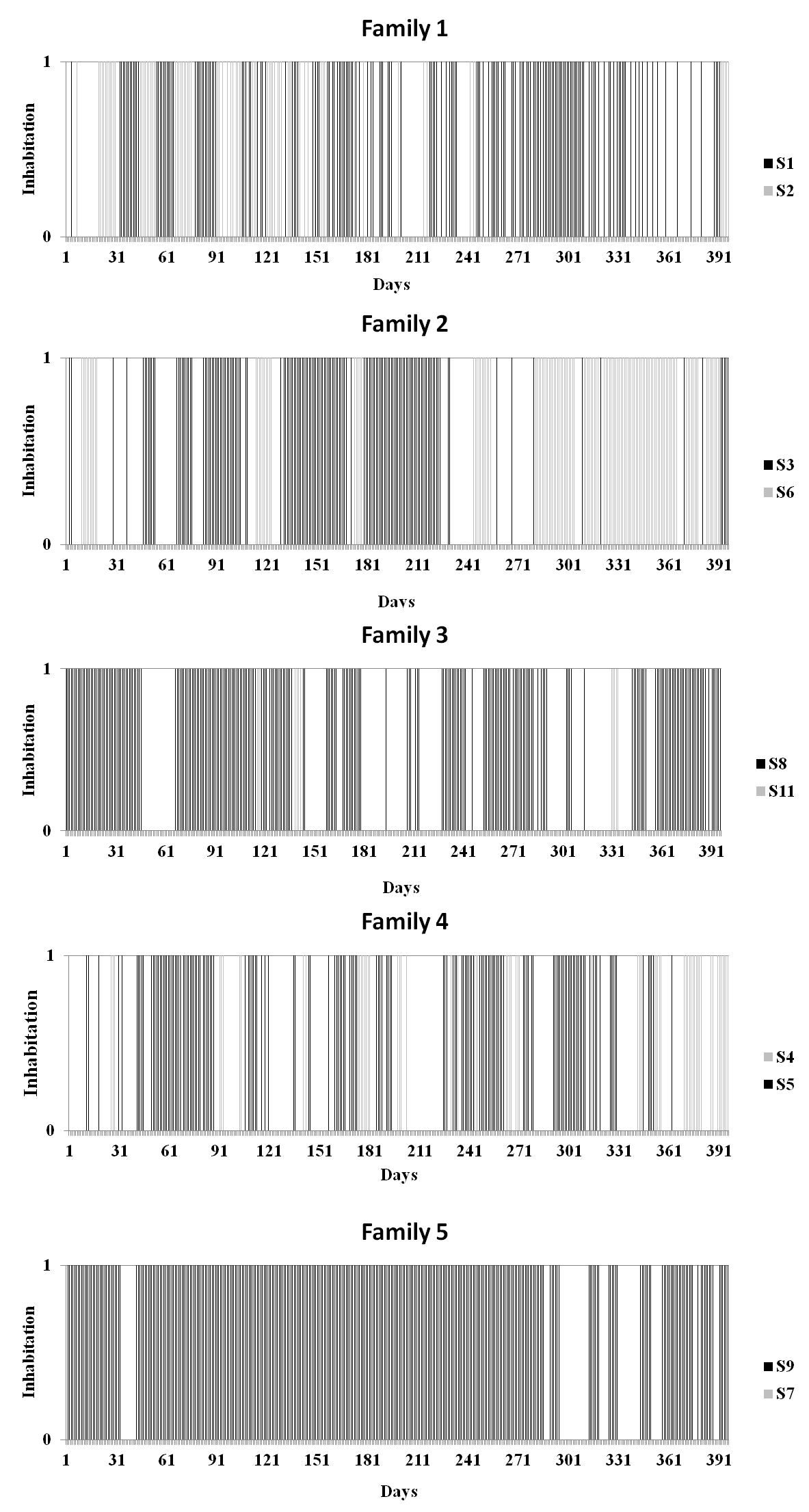

Supplement: Supplementary file 1 — Supplementary Figure S1. [file 41598_2022_9501_MOESM1_ESM.docx]
