# Supplementary material for: Spatio-temporal inhabitation of settlements by Hystrix cristata L., 1758
Source: Sci Rep. 2022 Mar 31;12:5426. doi: 10.1038/s41598-022-09501-5 (PMC8971386; doi:10.1038/s41598-022-09501-5)

**Supplementary Figure S2.** Inhabitation pattern of porcupine family 1 in settlement S1 and S2 in 2017 and 2018 (left) and of porcupine family 4 in settlement S4 and S5 in the period January-July in 2018 and 2019 (right). Black colour always refers to the mostly inhabited settlement. White spaces indicate the days in which it was not possible to assess inhabitation.


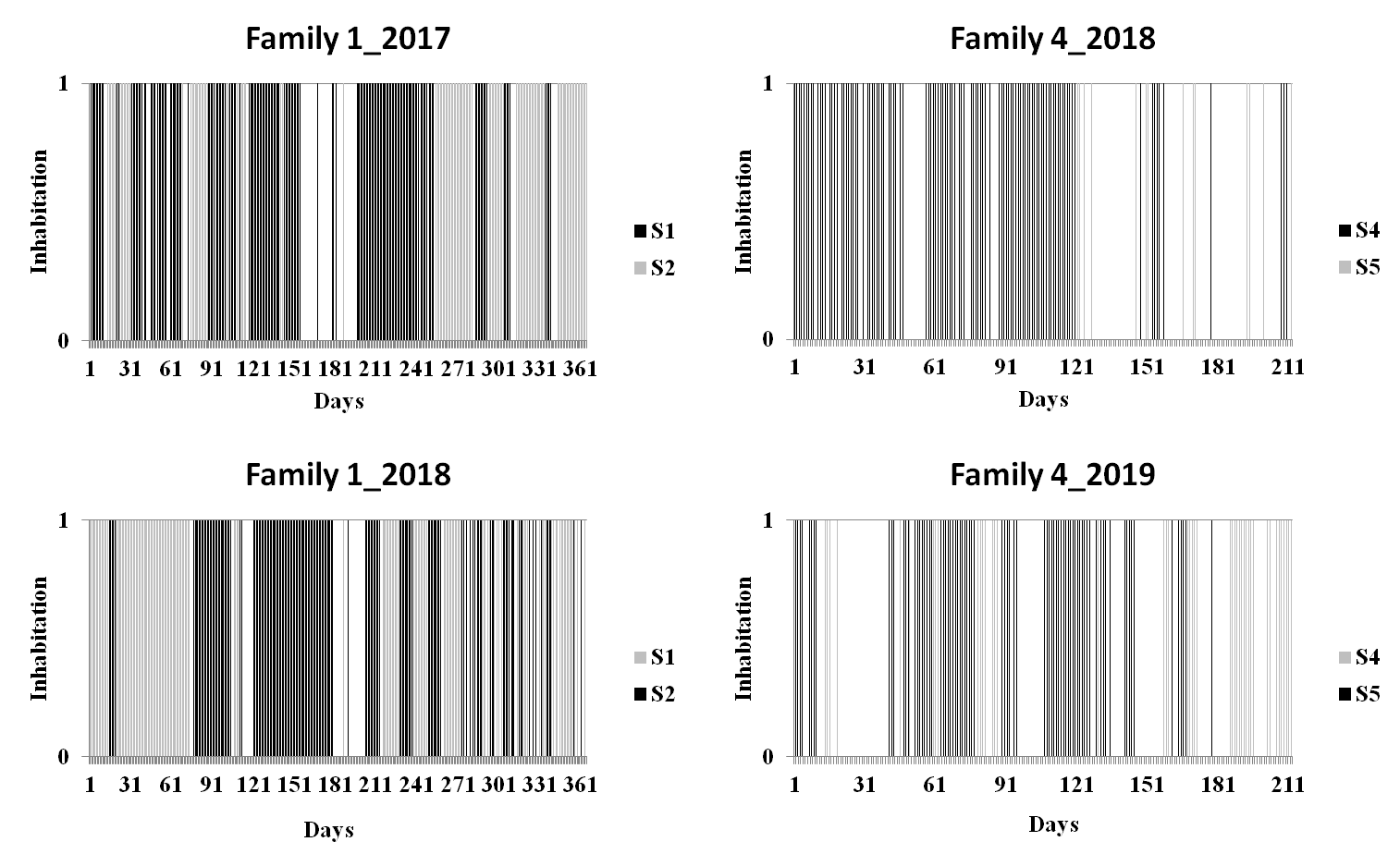

Supplement: Supplementary file 2 — Supplementary Figure S2. [file 41598_2022_9501_MOESM2_ESM.docx]
