# Supplementary material for: Spatio-temporal inhabitation of settlements by Hystrix cristata L., 1758
Source: Sci Rep. 2022 Mar 31;12:5426. doi: 10.1038/s41598-022-09501-5 (PMC8971386; doi:10.1038/s41598-022-09501-5)
Supplement: Supplementary file 3 — Supplementary Table S1. [file 41598_2022_9501_MOESM3_ESM.docx]

**Supplementary Table S1.** Porcupines individually marked and/or recognisable due to the presence of phenotypic peculiarities in each identified porcupine family. For the individuals phenotypically recognisable the phenotypic characteristic is reported**.**

|  | **Specimens** | **Marked** | **Recognisable** |
| --- | --- | --- | --- |
| **Family 1** | Adult male | X | White tapes on the quills  Blindness left and right |
|  | Sub-adult female | X | Red tapes on the quills  Black paint on the crest |
|  | Porcupette male | X | White paint on the tail |
| **Family 2** | Adult male | X | Black tapes on the quills  Blindness left |
|  | Adult female | NO | Blindness left |
|  | Porcupette female | X | White tapes on the quills |
|  | Sub-adult | NO | Absence of crest |
| **Family 3** | Adult female | NO | Blindness left |
|  | Adult male | NO | Blindness right |
| **Family 4** | Adult male | X | Black tapes on the quills  Black paint on the tail |
|  | Sub-adult female | X | Red tapes on the quills  White paint on the tail |
| **Family 5** | Adult male | X | White tapes on the quills Injury on the nose |
|  | Adult female | NO | Crest carried on the left side |
| **Family 6** | Sub-adult female | X | White tapes on the quills |
|  | Porcupette female | X | Black tapes on the quills |
|  | Sub-adult male | NO | Injuries in the rump (left) |
