# Supplementary material for: Spatio-temporal inhabitation of settlements by Hystrix cristata L., 1758
Source: Sci Rep. 2022 Mar 31;12:5426. doi: 10.1038/s41598-022-09501-5 (PMC8971386; doi:10.1038/s41598-022-09501-5)
Supplement: Supplementary file 4 — Supplementary Table S2. [file 41598_2022_9501_MOESM4_ESM.docx]

**Supplementary Table S2**: Settlement exposition, total number of holes and the monitored ones with the respectively monitoring time for each investigated settlement (S1 to S12).

| **Settlement** | **Orientation** | **N° holes** | **N° holes monitored** | **Monitoring time (months)** |
| --- | --- | --- | --- | --- |
| S1 | SW | 8 | 4^*^ | 31 |
| S2 | SE | 5 | 5 | 31 |
| S3 | SE | 4 | 4 | 16 |
| S4 | NW | 2 | 2 | 22 |
| S5 | SE | 5 | 5 | 22 |
| S6 | NE | 4 | 4 | 16 |
| S7 | SW | 12 | 4^*^ | 13 |
| S8 | NW | 5 | 5 | 13 |
| S9 | NW | 13 | 3^*^ | 13 |
| S10 | NW | 12 | 5^*^ | 10 |
| S11 | SW | 3 | 3 | 13 |
| S12 | SW | 16 | 5^*^ | 10 |

^*^ All ground holes were previously monitored in order to choose those

most used by crested porcupine for monitoring
